# Single-center experience of utilization and clinical efficacy of segmented leads for subthalamic deep brain stimulation in Parkinson’s disease

**DOI:** 10.1016/j.prdoa.2024.100273

**Published:** 2024-09-27

**Authors:** Ana Luísa de Almeida Marcelino, Viktor Heinz, Melanie Astalosch, Bassam Al-Fatly, Gerd-Helge Schneider, Patricia Krause, Dorothee Kübler-Weller, Andrea A. Kühn

**Affiliations:** a Movement Disorder and Neuromodulation Unit, Department of Neurology, Charité - Universitätsmedizin Berlin, Corporate Member of Freie Universität Berlin and Humboldt-Universität zu Berlin, Charitéplatz 1, 10117 Berlin, Germany; b Department of Neurology, Charité - Universitätsmedizin Berlin, Corporate Member of Freie Universität Berlin and Humboldt-Universität zu Berlin, Hindenburgdamm 30, 12200 Berlin, Germany; c Department of Neurosurgery, Charité - Universitätsmedizin Berlin, Corporate Member of Freie Universität Berlin and Humboldt-Universität zu Berlin, Charitéplatz 1, 10117 Berlin, Germany; d Berlin Institute of Health at Charité – Universitätsmedizin Berlin, Charitéplatz 1, 10117 Berlin, Germany; e Berlin Center for Advanced Neuroimaging, Bernstein Center for Computational Neuroscience, Berlin, Germany; f Exzellenzcluster NeuroCure, Charité - Universitätsmedizin Berlin, Berlin, Germany; g Berlin School of Mind and Brain, Humboldt - Universität zu Berlin, Berlin, Germany; h Deutsches Zentrum für Neurodegenerative Erkrankungen, Berlin, Germany

**Keywords:** Parkinson’s disease, Deep brain stimulation, Directional steering, Segmented leads

## Abstract

**Background:**

Segmented electrodes for deep brain stimulation (DBS) of the subthalamic nucleus (STN) in Parkinson’s disease (PD) enable directional current steering leading to expanded programming options.

**Objective:**

This retrospective study covering a longitudinal period of up to 7 years compares the efficacy of segmented and non-segmented leads in motor symptom alleviation and reduction of dopaminergic medication in PD patients treated in a specialized center and assesses the long-term use of directional steering in clinical routine.

**Methods:**

Demographic data and clinical scores before surgery and at 12-month follow-up (12MFU) as well as stimulation parameters at 12MFU and last follow-up (LFU) were assessed in all patients implanted with segmented leads between 01/2016 and 12/2019 and non-segmented leads in a corresponding time-period. Patients were classified as very good (>60 %), good (30–60 %) and poor (<30 %) responders according to DBS-induced motor improvement.

**Results:**

Clinical data at 12MFU was available for 61/96 patients with segmented (SEG) and 42/53 with non-segmented leads (N-SEG). Mean DBS-induced motor improvement and reduction of medication at 12MFU did not differ significantly between SEG and N-SEG groups or in a subgroup analysis of steering modes. There was a lower proportion of poor responders in the SEG compared with the N-SEG group (23% vs. 31%), though not statistically significant. At LFU, the percentage of patients set at directional steering increased from 54% to 70%.

**Conclusion:**

Efficacy in reduction of motor symptoms and medication does not differ between electrode types for STN-DBS at 12 months follow-up. The use of directional steering increases over time and may account for a lower proportion of poor responders.

## Highlights

• Segmented leads may broaden the therapeutic window of deep brain stimulation.
• STN-DBS led to a relevant improvement of motor symptoms regardless of lead type.
• Segmented electrodes may account for a lower number of poor responders to DBS.
• Directional steering can become more relevant in the course of the disease.

## 1 Introduction

Deep brain stimulation (DBS) of the subthalamic nucleus (STN) is an established treatment for Parkinson’s disease (PD) patients with (early) motor complications or severe tremor insufficiently controlled by medication [1], [2]. Specifically, the dorsolateral part of the STN should be targeted for efficacious motor symptom alleviation [3]. Unintended stimulation of adjacent fibers due to suboptimal electrode location or need for higher amplitudes in the course of the disease can cause adverse effects such as dysarthria, sensory disturbances, or muscle contractions. Conventional electrodes used for STN-DBS in PD consist of four ring contacts that allow omnidirectional stimulation around the circumference of each contact. Segmented leads have been available for over eight years enabling directional current steering for more specific targeting of the STN motor region. Although several studies have shown that this technology broadens the therapeutic window at short-term follow up [4], [5], [6], [7], studies proving increased efficacy in clinical outcome and long-term follow up are missing. One prospective cross-over, double blinded study over six months confirmed an enlarged therapeutic window in a cohort of > 200 PD patients [8] but did not show an advantage regarding the improvement of disease-specific motor symptoms. Increased use of directional steering over time has been demonstrated in a recent retrospective analysis of 56 PD patients at four timepoints postoperatively [9]. Clinical data at last follow-up (36 months) was available for nine PD patients showing significant reduction of post-operative dopaminergic medication but without data on motor outcome [9]. It is important to evaluate the benefits of this technological innovation for patient care, since contact selection with segmented leads is highly time-consuming in clinical routine. While clinical strategies for improving efficacy of programming have been suggested [10], automated programming algorithms, although promising, are not available for clinical use yet [11].

Our study aims to report on the experience with segmented leads and clinical outcome parameters in comparison with non-segmented leads at a large DBS center and provide first insights into long-term use of directional steering.

## 2 Methods

Medical records of all 96 PD patients who underwent STN-DBS surgery at our center with segmented leads between 01/2016 and 12/2019 (Boston Scientific Vercise Cartesia) were assessed. For comparison, we included all patients implanted with non-segmented leads at an equivalent time period (01/2013–12/2016 Boston Scientific Vercise Standard; 01/2015–01/2021 Medtronic 3389), yielding a total of 53 patients. Between 2016 and 2019 segmented leads were the standard at our hospital. In that period, only four patients received non-segmented leads (compatibility issues with cardiac pacemaker (1/4) or undocumented reasons (3/4)). Before that all patients received conventional leads as well as between 2020–2021, since a new IPG allowing for chronic sensing had been introduced before compatible segmented leads were available. Indication for STN-DBS surgery followed international standards [2].

All patients received bilateral STN implantation with exception of one patient in each group. One patient received STN and VIM electrodes, but the latter were not activated due to sufficient tremor suppression with STN-DBS. There was no change in surgical procedure including intra-operative microelectrode recordings and surgical team over the studied period. Demographic data, pre-operative clinical scores (UPDRS-III, levodopa equivalent daily dose [LEDD]) as well as post-operative clinical scores and DBS parameters at 12-month follow-up (12MFU; range 9–16 months post-operatively) and last follow-up (LFU; search limit 30th September 2023) were reviewed. At our center, a monopolar review is routinely performed three months after DBS implantation. Here, directional contacts will only be tested (and activated) if motor symptom alleviation is insufficient or limited by side effects. Electrode localization is used in individual cases when clinical programming is not straight-forward but does not per se trigger switching to directional steering.

Directional current steering was defined as an asymmetrical activation of the segmented contacts (> 5 % difference) on the horizontal plane in at least one of the pairwise implanted electrodes. Patients were included in the analysis if pre-operative and 12MFU UPDRS-III (Stimulation On/Medication Off and Stimulation Off/Medication Off) and LEDD scores were available in archival charts or accessible on video, leading to complete data sets for 61/96 patients in the segmented leads group (“SEG”, age 60 ± 1.3; 21 female) and 42/53 in the non-segmented group (“N-SEG”, age 59 ± 1.2; 11 female) with a total of 204 leads for final analysis.

Stimulation-induced motor symptom alleviation at 12MFU was calculated as (UPDRS-III_OFF_ – UPDRS-III_ON_)/ UPDRS-III_OFF_*100, whereas OFF and ON refer only to DBS (in both cases Off medication). Levodopa equivalent daily dose (LEDD) was calculated according to current guidelines [12] and reduction after DBS calculated as (LEDD_BL_ – LEDD_12MFU_)/LEDD_BL_*100. To evaluate the distribution of DBS-induced motor improvement, we stratified motor response into poor (<30 % improvement), good (30–60 %) and very good (>60 %), in line with previous literature of clinically meaningful treatment response in PD [13], [14]. Electrode localization was performed in poor responders with Lead-DBS (lead-dbs.org) according to established methods [15] and Euclidean distance from closest contact to a previously established “sweet spot” [16] was calculated as published before [17].

Data was tested for normal distribution using the Kolmogorov-Smirnov test. Clinical parameters were compared with paired parametric t-tests within and unpaired parametric t-tests (Welch’s test) between the SEG and N-SEG groups. ANOVA was applied when comparing the three stimulation modes (SEG_dir_, SEG_omni_ and N-SEG). Non-parametric baseline variables were compared using the Mann-Whitney test. For comparison of categorical variables, the χ2 test was applied. Statistical significance was set at p < 0.05. Results are presented as mean ± standard error of mean. This retrospective study was performed in accordance with the World Medical Association Declaration of Helsinki and was approved by the local Ethics Committee (EA1/264/23).

## 3 Results

### 3.1 Demographics

There was no significant difference in main demographic data between the SEG and N-SEG groups with regard to age, disease duration, PD subtype or levodopa equivalent dosage (see Table 1 for details). Even though baseline motor severity off medication was higher in the N-SEG group compared to SEG (UPDRS-III OFF med 51.9 vs. SEG 43.2 Pts.; Welch’s *t*-test p = 0.017), there was no significant difference on medication nor for the percentage reduction with levodopa between groups. In the SEG group, one patient had to undergo whole system explantation due to infection within the first three months postoperatively. Therefore, clinical parameters after re-implantation were considered for analysis. One patient eventually had his electrodes explanted and re-implanted due to misplacement – here, only the first implantation (including 12MFU and LFU) was considered in order to avoid bias.

**Table 1:** Baseline demographics of included patients in two groups according to the electrode type (“SEG”, segmented leads; “N-SEG”, non-segmented leads). For *, non-parametric tests were applied (see methods).

	**“SEG”**	**“N-SEG”**	
Patients (N)	61	42	
Age (yrs)	60 ± 1.3	59 ± 1.2	p = 0.6
Gender (N female)	21	11	p = 0.4
Disease duration* (yrs)	11.7 ± 0.7	10.5 ± 0.7	p = 0.3
PD subtype (equivalent/akinetic/tremor)	41/43/16 %	40/40/19 %	p = 0.9
Early-onset PD (N)	13 (21.3 %)	7 (16.7 %)	p = 0.6
UPDRS-III OFF/ON Medication (Pts.)	51.9/24.3	43.2/20.9	p = 0.017/0.14
L-Dopa induced Δ UPDRS-III* (%)	51.5 ± 2.5	52.6 ± 2.7	p = 0.95
LEDD* (mg)	1309.2 ± 61.9	1364.3 ± 69.5	p = 0.95

mean ± standard error of the mean.

### 3.2 Directional steering and motor symptom alleviation at 12MFU

Twelve months after surgery, both SEG and N-SEG groups reached a significant mean motor improvement in UPDRS III with STN DBS ON vs. OFF (SEG_ON-DBS_ = 28.02, SEG_OFF-DBS_ = 51.98 pts., paired *t*-test p < 0.0001; N-SEG_ON-DBS_ = 24.76, N-SEG_OFF-DBS_ = 44.17 pts.; p < 0.0001; OFF-medication). Percentage improvement with DBS did not differ between groups, with 45.3 % for SEG and 44.7 % for N-SEG at 12MFU (Welch’s *t*-test p = 0.88, t = 0.1396, df = 80.10). Furthermore, post-operative LEDD reduction was significant in both groups (pre-operative values in Table 1; 12MFU: SEG_LEDD_ = 470.07, p < 0.0001; N-SEG_LEDD_ = 484.76, p < 0.0001), but percentage decrease did not yield a significant difference between patients implanted with segmented and non-segmented leads (SEG 64.2 % vs. N-SEG 64.4 %; Welch’s *t*-test p = 0.96, t = 0.04419, df = 83.19).

**Fig. 1:**
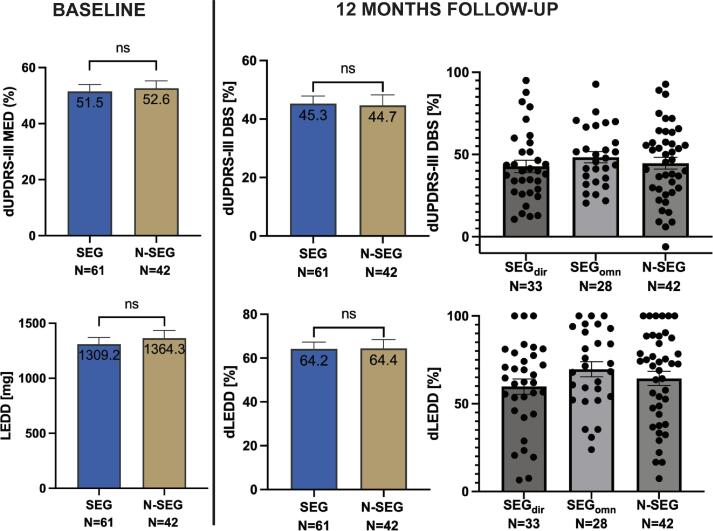
Main results. Baseline reduction of motor symptoms (UPDRS-III) with dopaminergic medication on the left and post-operative, DBS-induced, reduction of motor symptoms at 12MFU for both electrode types (SEG/N-SEG) and stimulation groups (SEG_dir_/SEG_omni_/N-SEG) on the right. Below, absolute levodopa equivalent daily dose at baseline (left) and pre- to post-operative reduction between groups (right). Color figure available online.

In the SEG group at 12MFU, 33 patients (58 ± 2.1 years, 10 female) were set with directional and 28 patients (62 ± 1.4 years, 11 female) omnidirectional steering parameters. These “sub-groups” will be referred to as SEG_dir_ and SEG_omn_. Disease duration until surgery comprised 12 ± 1.1 and 11.3 ± 0.75 years in the SEG_dir_ and SEG_omn_ groups at 12MFU, respectively. There was no difference in motor improvement with stimulation nor pre- to postoperative LEDD reduction between all groups (SEG_dir_, SEG_omn_, N-SEG) using ANOVA (dUPDRS-III p = 0.6, dLEDD p = 0.3). Fig. 1 shows mean values and distribution of data.

**Fig. 2:**
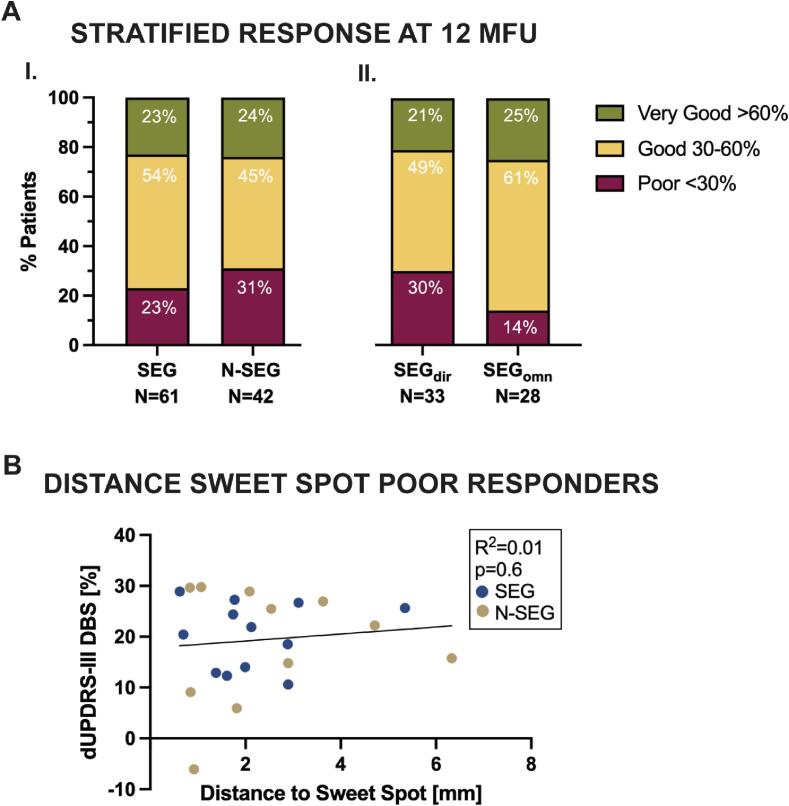
Stratification of DBS response. A: Stratified response of DBS-induced reduction in UPDRS-III at 12MFU given for electrode type (I.) (SEG vs. N-SEG) and stimulation setting within the segmented group (II.) (SEG_dir_ vs. SEG_omn_). B: Linear regression of Euclidean distance from closest contact to the “sweet spot” averaged across both hemispheres [16] and DBS-induced response of poor responders in SEG (12/14) and N-SEG (11/13) groups. Color figure available online.

Stratification of DBS-induced motor improvement revealed that the proportion of very good responders was above 20 % for both electrode types and all stimulation modes. Patients implanted with segmented leads (SEG) showed lower proportion of poor responders comparing with the non-segmented leads group (N-SEG; 23 % vs. 31 %) as depicted in Fig. 2A. Within the SEG group, the proportion of poor responders was lower in the omnidirectional stimulation group (14 % in SEG_omn_ vs. 30 % in SEG_dir_). Nevertheless, the distribution of motor responses did not differ significantly between electrode types or stimulation settings (Fig. 2A) as assessed using χ2 test (p = 0.6 for SEG vs. N-SEG; p = 0.3 for SEG_dir_ vs. SEG_omn_). A linear regression between the Euclidean distance from closest contacts to the “sweet spot” and DBS-induced motor outcome in all poor responders was not statistically significant (R^2^ = 0.01, p = 0.6) as depicted in Fig. 2B. A detailed assessment of medical records of poor responders in the SEG_omn_ group revealed that in all directional steering had been tried without added clinical value. Limiting factors for programming in these four patients included suboptimal electrode localization, a concomitant cardiac pacemaker and a complicated post-operative course with cognitive deterioration in a patient with heterozygous GBA mutation. However, these patients still showed an improvement over 20 %, as seen in Fig. 1.

### 3.3 Long-term use of directional steering increases for broader therapeutic window

Last follow-up was available for 59/61 patients in the segmented group and took place on average 52.5 ± 2.2 months after surgery (range 16–87 months). About 75 % of patients (44/59) maintained the stimulation settings at LFU (29/33 directional and 15/28 ring-mode stimulation, respectively). Changes in stimulation settings occurred mainly in patients initially set at ring mode. Specifically, 12 patients (44.4 %) of those had been switched to directional stimulation after 12MFU. The opposite (change from directional to ring mode after 12MFU) was only seen in 3/28 patients. Thus, the proportion of patients with directional steering increased from 54 % to 69.5 % from 12MFU to LFU. Reasons for switching from omnidirectional to directional current steering were gait disturbance (8/12), dysarthria (3/12), muscle contractions (1/12) and other stimulation-induced side effects (2/12). Full UPDRS motor score was not available at last FU. From the 59 patients, 4 patients had died at time of consultation of medical records for LFU: one due to suicide 24 months after surgery due to long-standing depression, one of septicemia after leg amputation due to peripheral artery disease, one from pneumonia and one at home for unknown reasons.

## 4 Discussion

In this study, we show in a large, single center cohort of PD patients that mean motor improvement at 12 MFU did not differ between patients with segmented or non-segmented electrodes and did not depend on directional steering. Similarly, reduction in LEDD did not show a significant difference between groups. However, the proportion of patients with poor motor response was smaller in the SEG cohort compared to N-SEG, although this did not reach statistical significance. Moreover, we showed an increased use of directional steering during long-term follow-up that was initiated to avoid stimulation side effects or improve motor symptoms as revealed by retrospective real-life data, pointing to an increased use of directional steering in selected patients at long-term.

Initial studies investigating the added value of segmented leads were performed intra- or shortly post-operatively and demonstrated a larger therapeutic window [5], [6], [7]. However, these and following larger retrospective studies did not show an advantage in the reduction of motor symptoms on a group level [6], [8], [9]. This is consistent with our results that show a highly significant DBS-induced motor improvement (∼41–43 %) but no difference between electrode types. Motor improvement and medication reduction (∼64 % in both groups) in our cohort are similar to results from DBS effects reported in clinical trials in PD [18]. This confirms an overall good clinical effect of STN DBS in our cohort.

Additional value of directional steering would be expected if electrodes were not perfectly placed in the “sweet spot” for STN DBS. Directional steering would allow to direct currents towards the target area and avoid stimulation of passing fibers thereby reducing side effects. Models using simulation of VTA (“volume of tissue activated”) suggest that current steering may account for optimizing misplacement of up to 1 mm depending on the rotation degree [19]. Thus, we expected that individually adjusted directional steering would decrease the number of patients with poor response and/or increase the percentage of patients with very good response. The relative smaller percentage of poor responders in the SEG group at 12 MFU found in our cohort is in line with the possibility to compensate for minor lead displacement using directional steering [19]. This is further supported by our observation of increased use of directional steering at long-term follow-up. However, findings may reflect and depend on further patient-specific factors (e.g. pre-operative l-dopa response, genetic mutations) as well as center-specific clinical practices in postoperative management of PD patients undergoing DBS that are independent of electrode localization.

Reasons to change from omnidirectional to directional steering included new motor symptoms that can be attributed to progress in neurodegeneration and higher stimulation amplitudes needed over time, resulting in side-effects when lead localization is suboptimal. Even though the increased use of directional steering observed in our cohort on the long-term supports this hypothesis, it should be explored in further prospective studies including more detailed long-term clinical data.

The question remains, how to take advantage of this technology in a time/cost-minimizing fashion in daily routine while awaiting implementation of automated programming algorithms in clinical practice. Debove and colleagues suggest that directional steering should be tested at the initial programming session when monopolar review in ring mode yields a side effect threshold ≤ 2 mA [10]. Eventually, this should be re-evaluated in the course of disease when side effects appear after increase in amplitude. Recently available DBS devices allowing chronic sensing could help to implement electrophysiological parameter for contact selection to improve programming and adjust it over the course of the disease [20]. Our study is limited by its retrospective nature and results reflect the clinical routine at our center, including in advanced programming. Furthermore, the difference in baseline motor severity off medication represents a potential bias, thus limiting generalizability of results. Lastly, reasons for changing parameter settings and their effect have not always been documented in detail.

## 5 Conclusion

This retrospective study corroborates previous findings that show preferred use of directional settings over omnidirectional stimulation already in the first year after implantation. However, no significant improvement in motor symptoms could be observed at the group level. Importantly, our results show that segmented leads may account for a smaller proportion of poor responders and that advantages of directional steering may become more evident in the long-term.

## CRediT authorship contribution statement

**Ana Luísa de Almeida Marcelino:** Writing – review & editing, Writing – original draft, Visualization, Methodology, Formal analysis, Data curation, Conceptualization. **Viktor Heinz:** Writing – review & editing, Formal analysis, Data curation. **Melanie Astalosch:** Writing – review & editing, Data curation. **Bassam Al-Fatly:** Writing – review & editing, Formal analysis. **Gerd-Helge Schneider:** Writing – review & editing, Data curation. **Patricia Krause:** Writing – review & editing, Supervision. **Dorothee Kübler-Weller:** Writing – review & editing, Supervision, Conceptualization. **Andrea A. Kühn:** Writing – review & editing, Supervision, Methodology, Conceptualization.

## Declaration of competing interest

The authors declare the following financial interests/personal relationships which may be considered as potential competing interests: [A.L.A.M., V.H., M.A., B.A. and DKW have no conflicts of interest to declare. PK received speakeŕs honoraria from Stadapharm, AbbVie and MedTronic outside of this work and is in the Advisory Board of Abbvie and MedTronic. G.-H.S. has received honoraria from Medtronic, Boston Scientific, and Abbott unrelated to this work. A.A.K. has served on advisory boards of Medtronic and has received honoraria and travel support from Medtronic, Boston Scientific and Stada Pharm outside of this work].
